# Optimization and Application of Communication Resource Allocation Algorithm for Urban Rail Transit Planning

**DOI:** 10.1155/2022/5608665

**Published:** 2022-08-25

**Authors:** Hui Fang, Wei Zhang

**Affiliations:** ^1^Zhejiang University, Hangzhou, Zhejiang 310027, China; ^2^Ningbo Rail Transit Group Co., Ltd, Ningbo, Zhejiang 315101, China

## Abstract

The construction and operation of China's rail transit system have entered a high-speed development stage, and the rapid increase of train speed and mileage has brought greater challenges to the safety and reliability of the rail transit system. Network planning evaluation is the key to the early decision-making of urban rail transit project, which directly determines the success or failure of the whole project. How to scientifically and reasonably evaluate the urban rail transit information resource network planning has become a difficult problem for many urban planners to solve. Therefore, this paper studies the optimization of the communication resource allocation algorithm and the comprehensive evaluation of its application for urban rail transit planning. In this paper, based on CVNN structure, the network prototype is an extension of RVNN structure. In the abstract, its processing unit is composed of a pair of real-number processors that can realize certain operations. HNN is a fully connected recurrent neural network based on the idea of the energy function, which is helpful to understand the calculation mode of HNN, and the research shows that HNN can solve many combinatorial optimization problems. In addition, the combination of neural network and genetic algorithm with simulated annealing mechanism can also bring new directions for research. On the basis of experimental analysis, it can be concluded that in general, the error reduction rate of the optimization scheme designed in this paper can reach 58.6% on average. In practical application, the accuracy of the optimal bit error rate is 52.4%.

## 1. Introduction

Urban rail transit has the characteristics of large volume, fast speed, safety and convenience, and high reliability and has become the main mode of transportation to relieve urban traffic pressure [1]. Under normal conditions, the train runs in strict accordance with the operation diagram. Urban rail transit network planning refers to the activities that the government planning department, after reasonably studying the relevant national strategic objectives and analyzing the social and economic development of the region in a certain period of time, determines the construction of rail transit according to the development objectives of the overall urban planning, analyzes the local traffic demand, the scale and layout of the network, and comprehensively arranges the traffic planning and carries out the comprehensive deployment of urban rail transit construction on the basis of rationally and effectively utilizing urban land and coordinating the functions, urban space, and layout of various traffic modes [2]. In the 21st century, China's economic development has made remarkable achievements, and the urban scale has expanded unprecedentedly [3]. However, the accompanying crises such as natural disasters, population growth, infectious diseases, fires, food shortages, ecological destruction, resource crises, environmental pollution, and even terrorist incidents threaten the healthy development of cities, especially urban traffic problems, road congestion, traffic jams, and traffic disorder are particularly prominent, which together constitute an “urban crisis.”

Multipath fading and time-varying are the most basic characteristics of wireless channels, which makes the channel fading among users very different even at the same time [4]. In order to prevent the same frequency interference effect, the same channel is not allowed to be occupied by multiple users. Therefore, the dynamic subcarrier allocation algorithm must be used to reasonably allocate subcarriers according to the instantaneous channel information of each user subcarrier [5]. At the same time, as an important part of urban overall planning, it should be organically integrated with urban functional zoning, urban land development and urban design, and urban environmental ecological protection [6]. China's cities are highly motivated to build rail transit projects, but they often lack thoughtful, scientific, and reasonable rail transit network planning, and have insufficient knowledge of the types and characteristics of modern rail transit. Therefore, the choice of rail transit types is subjective and one-sided [7]. Urban rail transit network planning evaluation runs through the line network scheme design and optimization decision-making. As one of the key links of rail transit planning, it directly determines the quality of urban rail transit network planning, which is deeply related to the success or failure of the whole urban rail transit system [8]. Therefore, it is absolutely necessary to evaluate the urban rail transit network planning scientifically and effectively, and it has profound practical significance [9]. At present, most of the research work is focused on the resource allocation of complete channel information. However, in the actual system, the user's channel state information cannot be completely error-free fed back to the base station, and there may be channel estimation error, channel feedback delay, channel feedback error, and so on. Therefore, it is necessary to study the resource allocation under incomplete channel information and design a more reasonable resource allocation algorithm.

A genetic algorithm is a kind of bionic heuristic algorithm simulating biological evolution theory. It is an optimization method realized by simulating the selection genetic mechanism of “survival of the fittest” in nature [10]. The generation process is as follows: randomly generate a batch of populations according to the scale of the problem, and the populations go through three operation processes of random selection, crossover, and mutation to eliminate individuals with low fitness and retain individuals with higher fitness. The number of iterations and continuous reproduction and evolution, and finally get a group of individuals that are most adapted to the environment, and do certain processing to the optimal individual to obtain the solution to the problem. Artificial neural network is a mathematical model based on the human brain nervous system, which is composed of a large number of neurons, including dendrites and axons [11]. Dendrites are used to collect information from other nerve cells, cell bodies process the information collected by dendrites, and axons are used to transmit the information brought by cell bodies [12]. At present, the state monitoring of the rail transit system mainly relies on the combination of manual inspection, comprehensive monitoring of train inspection, and on-board inspection equipment to conduct “regular” inspection of the operation state of the rail transit system, and offline processing and analysis of the inspection data. The efficiency is closely related to the frequency of detection [13]. As the detection frequency is generally set according to the operation experience of field experts, there is a strong lag in the monitoring of sudden failures [14]. There are still deficiencies in the above research, so this paper puts forward the following innovations to study the optimization and application of communication resource allocation algorithm for urban rail transit planning:

① An optimization function is designed to update and optimize the sample, fully considering the influence of the sample size and the corresponding relationship of the components on the system energy consumption, to ensure the minimization of the overall energy consumption and the balance of the energy consumption of each node. Finally, a heuristic algorithm based on is used to optimize the clustering results, so that the subnet monitoring information can be transmitted under the optimal clustering routing protocol. According to the shortcomings of traditional complex valued BP neural networks, an improved CVNN is proposed. Combined with Kalman algorithm and wavelet concept, a complex-valued Kalman filter neural network and new complex-valued wavelet neural network are proposed, respectively.
② An energy-efficient criterion-based power minimization scheme and a genetic simulated annealing algorithm-based scheme are proposed to solve [15]. Based on the idea of optimal bit loading, the subcarrier number, subcarrier number, and bit number of users are adjusted optimally to minimize the power of the system. Then, by adding the simulated annealing selection mechanism to the genetic algorithm, it alleviates the selection pressure to a certain extent, overcomes the characteristics that it is easy to fall into local optimization, makes the algorithm converge quickly, and finds an approximate global optimal solution.

The chapters of this paper are arranged as follows: Section 1 of this paper is the introduction, which discusses the background and significance of the topic selection of the paper, and expounds the innovation points of the paper. Section 2 of this paper mainly combines the research results in the field of optimization and application of communication resource allocation algorithm for urban rail transit planning at home and abroad and proposes innovative results and research ideas of this paper. Section 3 of this paper is the method part, which deeply discusses the application and principle of related algorithms and puts forward the optimization and application model of the communication resource allocation algorithm based on the previous research results and the innovation of this paper. Section 4 of this paper mainly discusses the experimental part of the application of the algorithm. Through the experimental results, on the basis of sorting out the data, an optimization model is established. Section 5 is the summary part, which summarizes the research results of this paper.

## 2. Related Work

Pu and others believe that in the next ten years, the bandwidth demand of wireless communication will increase to above, and the currently allocated spectrum bandwidth resources can no longer meet the needs of mobile communication development. Therefore, it is very important to use the currently allocated spectrum resources more effectively [16]. Ke et al. put forward that in order to prevent the degradation of individual artificial fish in swimming, the behavior of artificial fish should be improved as follows. When the best artificial fish in the population performs foraging, chasing after the tail, and swarming, if the target position is not as good as its own position, the artificial fish will not move [17]. The research of Llerena and Gondim shows that D2D users use resources orthogonal to Cu users to communicate on D2D direct link. In this case, there is no interference between D2D users and Cu users, and the QoS of users is easier to be guaranteed. However, this does not fully take advantage of the benefits brought by D2D communication, such as multiplexing gain [18]. Chien and others believe that due to the relative position change of the transmitter and the receiver, the actual signal frequency received by the receiver will be shifted to some extent, resulting in a serious distortion of the received signal. How to solve this kind of problem, one of the key points is to establish a mobile communication channel model that is consistent with the actual channel [19]. Ma and others' research shows that the train of urban rail transit system is transported by randomly collected urban residents and floating population, which determines the complexity of urban rail transit transportation organization, and many random factors will inevitably interfere with the operation process, resulting in the actual operation of the train deviating from the planned operation diagram [20]. Mousumi and others believe that urban rail transit network planning is an extremely complex system engineering involving many factors, cross expertise, and huge investment. It is necessary to apply the principles of system science and combine the internal and external environment to systematically analyze the factors at all levels of “point,” “line” and “area” in order to obtain comprehensive and scientific research results [21]. Jamshidi et al. proposed that the communication energy consumption of nodes in the wireless sensor network for rail transit condition monitoring is jointly determined by the distance of information transmission and the amount of data, and data fusion processing at the cluster head node can effectively reduce the data consumption. The transmission volume and the corresponding communication energy consumption will increase the corresponding data processing energy consumption. At the same time, the change of data fusion rate will affect the energy efficiency of nodes in the process of data fusion processing and transmission, and then affect the life cycle of cluster head nodes [22]. Park et al. think that with the rapid increase of the number of smart devices, the amount of data transmission has also exploded, which also requires the continuous development of mobile wireless communication technology to meet people's growing business needs. In order to cope with the explosive growth of data traffic, a large number of device access, and the emergence of various new services, the fifth generation mobile communication system will soon appear in people's lives [23]. The research of Pasandideh and others shows that it is very necessary to pay attention to the research on urban rail transit network planning and the research on the optimization decision-making method of urban rail transit network planning, do a good job in a theoretical basis and make technical reserves for urban rail transit. Aiming at the multicriteria and multiobjective decision-making problem, it is of great significance to study the systematic optimization decision-making method of urban rail transit network planning and provide decision support for decision-makers in theory and application [24]. Dastgheib et al. designed a three-dimensional resource allocation method of power-mode-channel, but did not consider the cooperative mode in the mode selection problem. Among the three transmission modes, the cooperative mode has the highest spectral efficiency and also consumes the most power. From the perspective of optimizing energy efficiency, the cooperative mode should be considered together with the direct transmission mode and the two-hop mode [25]. Xu and others think that the number of cycles required for network training is determined by experiment and error. Too many training cycles often lead to the phenomenon of overtraining. Theoretically, overtraining will lead to error approximation. However, overtraining often leads to a serious decline in the generalization ability of test data. At the beginning, the error of the test part will decrease continuously in a certain number of training cycles [26]. Ni and Wang believe that the activation function of the neural network can limit the output oscillation of neurons. In order to determine the activation intensity of neurons, the input weighted signals of all neurons must be converted. By comparing the activation output and broad value of neurons, some functions are designed to represent the activation or inhibition state of meridians [27].

Based on the research of the above-mentioned related work, this paper determines the positive role of urban rail transit planning in the field of communication resource allocation algorithm optimization and application and builds an algorithm-optimized communication resource allocation algorithm optimization and application model. Data use big data algorithm analysis to conduct in-depth analysis and research, make more effective use of data, mine valuable knowledge hidden behind data, and discover and find potential problems that affect the optimization and application of communication resource allocation algorithms.

## 3. Methodology

### 3.1. Analysis and Research on Related Theories

#### 3.1.1. The Framework of Urban Rail Transit Planning

China's rail transit system has the characteristics of a large-scale operation network, high density, large carrying capacity, complex operation environment, fast operation speed, long continuous operation distance, and operation across climatic zones and geological environments. At the same time, rail transit is a complex linkage system, and local hazards may spread to the whole network, and even lead to the paralysis of the whole rail transit network. Rail transit network planning is an integral part of the city's overall planning and one of the most important special plans. The planning process must implement the strategy and development requirements of the city's overall planning, and support the city's geography, culture, economy, and land scale. Realization of development and structural adjustment to form a reasonable urban layout. Under the guidance of integrity, scientificity, operability, economy, foresight, and dynamics, we will guide the harmonious development of cities. Generally speaking, the factors affecting the macro level of urban rail transit network planning mainly include: national policies and strategies, economic development level, rail transit technology level, and environmental protection. The micro-level is related to the city itself, mainly including the form, scale, and planning of the city, the traffic demand of the city, the characteristics of residents' travel, and land use. Generally speaking, the train runs strictly according to the train plan. The interference of many random factors makes it inevitable that the train deviates from the plan and causes the running disorder. Therefore, a train operation adjustment model with a relatively broad sense is adopted:

(1) $$\begin{array}{c} G\left(j+1\right)=G\left(j\right)+T\times G\left(j\right). \end{array}$$

**Figure 1:**
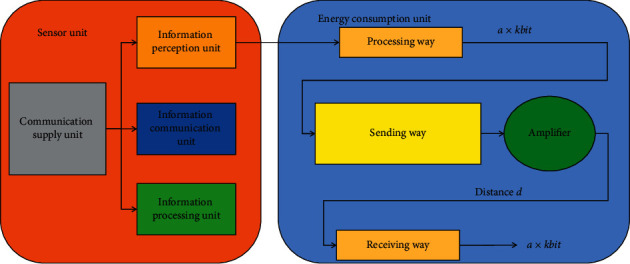
Composition and energy consumption structure of wireless sensor nodes.

*j*=0,1,…, *n*, *G*(*j*) are the actual running state of the train at time *j*, and *T* is the state transition parameter determined by the train operation adjustment decision. Among them, the sensor is an important component for controlling the train behavior, and it is also a data acquisition way to study the traffic communication resources.  Figure 1 shows the composition and energy consumption structure of wireless sensor nodes.

The sensor node usually consists of four parts: information sensing unit, information processing unit, information communication unit, and energy supply unit. Among them, the information sensing unit is responsible for sensing the real-time service status information of the monitored object; the information processing unit is responsible for processing such as simple format conversion of the sensing information. The information communication unit is responsible for information communication with other sensors or sink nodes, including the process of receiving and sending information. The energy supply unit is responsible for supplying energy to the other three units to ensure the stability of each unit.

The energy consumption of the sensor nodes in the subnetwork in the data communication process is mainly composed of three parts: information processing energy consumption, information receiving energy consumption, and information sending energy consumption. Among them, the information processing energy consumption is mainly determined by the information processing energy consumption rate and the amount of processed data, as shown in the following formula:

(2) $$\begin{array}{c} E_{DX}\left(l\right)=l\times E_{DA}, \end{array}$$

where *l* is the amount of data processed, and *E*_*DA*_ represents the energy consumed by the information processing circuit for processing the unit amount of data. General data receiving energy consumption is mainly determined by the amount of data received, and the expression is as follows:

(3) $$\begin{array}{c} E_{rx}\left(l\right)=l\times E_{\text{elec}}. \end{array}$$

In the previous formula, *l* is the number of data bits sent or received, and *E*_elec_ represents the energy consumed by the transmitting circuit.

#### 3.1.2. Communication Resource Allocation Algorithm

**Figure 2:**
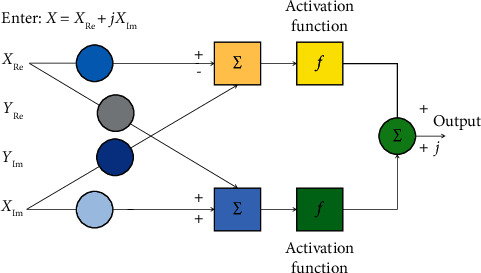
Single complex-valued neuron model.

CVNN structure is the network prototype of the extension of RVNN structure. In the abstract, its processing unit is composed of a pair of real-number processors that can realize certain operations. In this sense, they are minimal models of multidimensional neural computation, and the single complex-valued neuron model is shown in Figure 2.

The biggest advantage of CVNN is not the two-dimensional characteristics of the complex plane, but the increase of its synaptic weight, which is the initial progress made by the synaptic weight. CVNNs form modulation, phase rotation, and amplitude increase/decrease of two types of signal entities. The function of the whole neural network includes phase rotation and amplitude modulation, which makes CVNN less free of learning and self-organization than two-dimensional RVNN.

HNN is a fully connected recurrent neural network based on the idea of the energy function, which is helpful to understand the calculation mode of HNN, and the research shows that HNN can solve many combinatorial optimization problems. In the MA assignment problem, each element in the subchannel assignment matrix 8 can be HNN mapped to a neuron in the curve, and when the HNN iteratively converges, the output matrix of the network is the final subchannel assignment matrix.  Table 1 shows the subchannel allocation mapping of users.

Therefore, *K* × *N* neurons are needed to construct HNN in this algorithm to solve the MA optimization problem. The output of the second neuron is defined as

(4) $$\begin{array}{c} v_{i}=g\left(u_{i}\right)=g\left(\sum_{j=1,j\neq 1}^{N_{0}}w_{ij}v_{j}+b_{i}\right), \end{array}$$

where *w*_*ij*_ is the connection weight between the *j* th neuron and the *i* th neuron, *u*_*i*_, *v*_*i*_ are the input and output signals of the *i* th neuron, *b*_*i*_ is the threshold of the *i* th neuron, and *g*(·) is the neuron activation function.

In the HNN optimization problem, it is necessary to construct the energy function in order to ensure that the obtained state is stable. The energy function of HNN is actually a Lyapunov function. HNN adopts a gradient descent algorithm on the Lyapunov function until all neurons enter their own stable state. The general energy function maximization or minimization problem is often transformed into finding the differential state of the neuron output state, so the dynamic function of HNN can be described as

(5) $$\begin{array}{c} \frac{\partial E\left(u_{i},v_{i}\right)}{\partial u_{i}}=-\frac{\text{d}u_{i}}{\text{d}t}. \end{array}$$

With the previous formula, HNN can be applied to specific optimization problems.  Table 2 assigns the weight of each indicator to the plan.

Genetic algorithm is an adaptive intelligent optimization algorithm based on the concept of “survival of the fittest” in biological evolution. It is a general algorithm framework. It does not depend on any specific problem and is superior to the traditional optimization algorithm in powerful, large, and complex nonlinear systems. Simulated Annealing Algorithm Regardless of the initial selection value, the simulated annealing algorithm has the characteristics of being independent of the initial selection value and asymptotically converging. It has been proved theoretically that it is an optimization algorithm that approaches 1 with probability and converges to the global optimal solution. A genetic algorithm and simulated annealing algorithm are combined to make effective use of them, which makes the algorithm converge quickly and find the optimal solution of the problem.

### 3.2. Optimal Design Based on Communication Resource Allocation Algorithm

The traditional complex-valued BP algorithm not only does not improve the error signal of the output neuron, but also reduces the propagation performance of the error signal, and the differentiation of the activation function will cause a delay in the convergence of the algorithm. In addition, any saturation response function inevitably has the above properties, that is, its differential will not exist at the saturation point. In order to avoid the error caused by factors in the traditional complex valued BP algorithm, data processing is required to correct it. Therefore, by replacing the difference between the actual output and the ideal output, this section proposes the following minimum correction error function:

(6) $$\begin{array}{c} E_{p}=\frac{1}{2}\sum_{k=1}^{M}\left[D_{pk,R}{\text{Inf}}_{k}^{O}\left({\text{net}}_{pk,R}^{O}\right)+\left(1-D_{pk,R}\right)\text{In}\left(1-f_{k}^{O}\left({\text{net}}_{pk,R}^{O}\right)\right)\right]+\frac{1}{2}\sum_{k=1}^{M}\left[D_{pk,I}{\text{Inf}}_{k}^{o}\left({\text{net}}_{pk,I}^{o}\right)\;+\;\left(1-D_{pk,I}\right)\text{In}\left(1-f_{k}^{o}\left({\text{net}}_{pk,I}^{o}\right)\right)\right], \end{array}$$

where *M* represents the total number of neurons in the output layer and *f*_*k*_^*o*^(net_*pk*,*R*_^*o*^), *f*_*k*_^*o*^(net_*pk*,*I*_^*o*^) represent the real and imaginary parts of the actual output of the *k* th output neuron, respectively. *D*_*pk*,*R*_, *D*_*pk*,*I*_ are the real and imaginary parts of the ideal output of the *k* th output neuron. At the same time, the back-propagation of errors will also change, while the forward-propagation remains unchanged.

Wavelet is a new powerful tool to express nonlinearity. The function *f*(*x*) can be represented by the superposition of subwavelets *ψ*(*x*) of a mother wavelet *ψ*_*a*,*b*_(*x*). Where *ψ*_*a*,*b*_(*x*) can be expressed as

(7) $$\begin{array}{c} \psi_{a,b}\left(x\right)=\frac{1}{\sqrt{a}}\psi \left(\frac{x-b}{a}\right). \end{array}$$

At this time, the approximate expression of the function can be obtained:

(8) $$\begin{array}{c} f\left(x\right)\approx \sum_{k=1}^{K}w_{k}\psi \left(\frac{x-b_{k}}{a_{k}}\right). \end{array}$$

Among them, *w*_*k*_, *b*_*k*_, *a*_*k*_ represent the weight coefficient, expansion coefficient, and scale translation coefficient of each subwavelet, respectively. In this paper, *P*=[*P*_1_, *P*_2_ … *P*_*N*_]^*T*^ is used to represent the power allocation of all subcarriers, and *P*_*n*_ represents the power allocated to the *n* th subcarrier, then the rate of user *m* can be expressed as

(9) $$\begin{array}{c} R_{m}=\sum_{n=1}^{N}\frac{c_{mn}}{N}{\text{log}}_{2}\left(1+\frac{P_{n}h_{mn}^{2}}{N_{0}B/N}\right). \end{array}$$

In order to express the balance, this paper uses the following formula:

(10) $$\begin{array}{c} f_{\text{Fairness}}={\sum_{m=1}^{M}\left(R_{m}-\alpha_{m}\left(\frac{\sum_{m=1}^{M}R_{m}}{\sum_{m=1}^{M}\alpha_{m}}\right)\right)}^{2}. \end{array}$$

**Figure 3:**
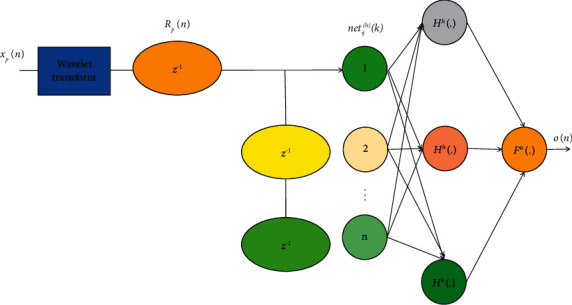
Embedded wavelet neural network structure.

The larger the *f*_Fairness_, the worse the fairness between users. The smaller the *f*_Fairness_, the better the fairness between users. The general approximate model representation is shown in Figure 3.

The initialization of the population adopts the initialization method of controlling the breeding range, and controls the breeding boundary according to the lag of the adjustment plan relative to the original plan, so that the time to initialize the corresponding position of the chromosome is not less than the original plan time and not greater than the total delay time. According to the general rule, at the temperature *T*, the probability of a particle tending to balance is *e*^−Δ*E*/(*kT*)^, where *E* is the internal energy at the temperature *T*, Δ*E* is its change, and *k* is a constant. Using solid annealing to simulate the combinatorial optimization problem, the internal energy *E* is simulated as the objective function value *f*, and the temperature *T* is evolved into the control parameter *t*, and the simulated annealing algorithm for solving the combinatorial optimization problem is obtained.

At this point, a discrete information bit loading concept is introduced: information granularity. It refers to the minimum information increment interval loaded by each subchannel in channel transmission, that is, the information bits transmitted on the actual channel must be incremented in units of information granularity, which is an integer multiple of the information granularity. It can be expressed as

(11) $$\begin{array}{c} b_{n}=N\beta . \end{array}$$

Among them, *n*=1,2,…, the general value of *β* is also 0.25, 1, 2, which are relatively simple values. In order to get the optimal scheme, this paper puts forward the effective criterion of bit distribution energy: suppose *b*={*b*_1_, *b*_2_ … *b*_*N*_} represents the bit distribution vector of *N* parallel subchannels, *E*(*b*_*n*_) is the bit energy loaded in the *n* channel, *β* is the information bit loaded every time, and remember *e*(*b*_*n*_)=*E*(*b*_*n*_) − *E*(*b*_*n*_ − *β*). If the bit distribution in *N* parallel subchannels satisfies

(12) $$\begin{array}{c} \underset{n}{\text{max}}\left[e\left(b_{n}\right)\right]\leq \underset{m}{\text{min}}\left[e\left(b_{m}+\beta \right)\right]. \end{array}$$

It indicates that the channel has achieved effective bit allocation, and the bit distribution in the channel has reached the best state. No bit exchange can reduce the total energy in the channel.

## 4. Result Analysis and Discussion

**Figure 4:**
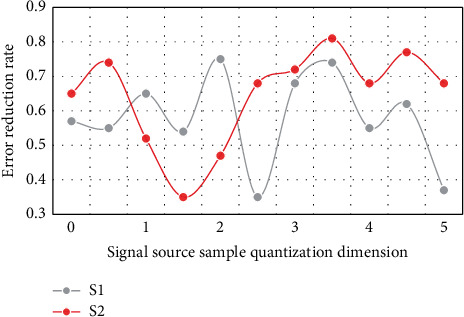
Error performance analysis.

**Figure 5:**
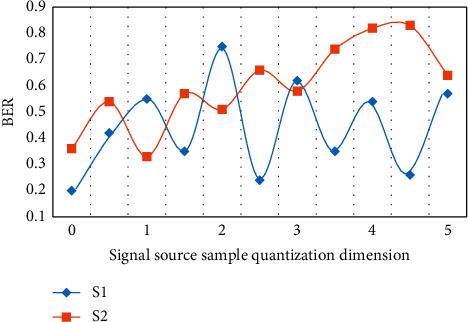
BER performance detection analysis.

In the above analysis, the optimization and application of the communication resource allocation algorithm for urban rail transit planning proposed in this paper are based on the combination of genetic algorithm and simulated annealing algorithm and neural network algorithm. In order to verify the rationality, scientificity, and feasibility of the scheme, this paper will conduct experimental analysis, and get the effect and practical application of the optimized scheme based on data analysis. For the accuracy of experimental results and analysis conclusions, the experiment follows the basic principles of unity and standardization. This paper will analyze and process from three important aspects: error performance, BER performance detection, and efficiency comparison between optimization algorithm and single algorithm. The following is the analysis and experimental data diagram of sample sets *S*1 and *S*2 in error performance and BER performance detection, as shown in Figures 4 and 5.

**Figure 6:**
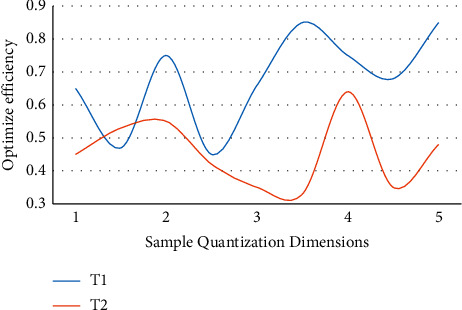
Comparison of the efficiency of the optimization algorithm and a single algorithm on different sample sets.

Through the above numerical analysis, it can be seen that the optimization algorithm designed in this paper has a good performance in error performance. There is good stability on the entire horizontal axis, and the error reduction is basically obvious, which has a good effect on the optimization and application of the communication resource allocation algorithm. Because there are interference items to be eliminated in the communication, the two sample sets basically keep the same decreasing trend in the 3-4 interval, but there are periodic changes, so they will return to the normal level after the next band. In general, the average error reduction rate of the optimization scheme designed in this paper can reach 58.6%. In the performance detection of BER, after the detection is implemented on the same sample set, it can be found that the overall trend is wavy and the amplitude is basically the same. Based on the improved neural network algorithm, the approximate optimal bit error rate can be obtained by optimizing the allocation of communication resources, which is far superior to the traditional allocation algorithm in performance. In practical application, the accuracy of the optimal bit error rate is 52.4%.  Figure 6 shows the efficiency comparison between the optimization algorithm *T*1 and the single algorithm *T*2.

It can be seen from the experimental data that there are obvious differences between the optimization algorithm and the single algorithm efficiency in different sample sets, and this difference also leads to the difference in the final results. Generally, the improvement of efficiency is an important parameter that needs to be considered in a scheme, model, or system. In the optimization scheme of embedded genetic algorithm, based on the characteristics of mutation and crossover, a breakthrough is made in the algorithm structure. Because of the good processing ability of bit distribution, the overall processing efficiency will be higher. The overlap in the range of 1-2 is caused by the accumulation of interference items due to the lack of samples in the early stage. With the expansion of the content of samples, the influence of interference items will be weakened under the differentiation and processing of algorithms. Therefore, after stages 3-4, the optimization algorithm will be significantly higher than a single algorithm.

## 5. Conclusions

The development and improvement of rail transit is a symbol of the strength of a city. It can not only make the city's traffic layout more reasonable, ease the increasingly congested traffic environment, and improve urban efficiency, but also promote balanced urban development and accelerate the process of urban integration. A reasonable rail transit network can bring into play the huge economic and social benefits of the city, and it is an important factor affecting the development of the structure and function of big cities. The realization of urban rail transit function is not only the result of planning, but also the result of decision-making. Based on the analysis of the neural network algorithm, genetic algorithm, and simulated annealing mechanism, this paper further studies the improvement of CVNN, and puts forward a new CVNN algorithm and optimization scheme. In order to reduce the complexity, the number of subcarriers is allocated first, and then the subcarrier allocation and power allocation are performed. The number of subcarriers is allocated according to the proportional rate requirements of each user, and the subcarriers with better channel state information are searched for each user according to the number of subcarriers. The number of subcarriers is allocated proportionally according to the rate demand and adjusted according to the proposed energy efficiency criterion. Then, the corresponding optimal subcarriers are found horizontally and vertically from the channel state information matrix. Finally, each user first distributes bits evenly on the allocated subcarriers, and then makes the optimal adjustment according to the energy efficiency criterion. On the basis of experimental analysis, it can be concluded that in general, the error reduction rate of the optimization scheme designed in this paper can reach 58.6% on average. In practical application, the accuracy of the optimal bit error rate is 52.4%.

## Figures and Tables

**Table 1:** Subchannel allocation mapping of users.

	*N*1	*N*2	*N*3
User1	1	1	0
User2	0	0	0
User3	0	1	1

**Table 2:** Planning to assign the weight of each indicator.

Factor	Weights	Factor	Weights	Factor	Weights	Factor+	Weights
*X*_11_	0.1078	*X*_21_	0.0127	*X*_31_	0.1574	*X*_41_	0.0354
*X*_12_	0.0145	*X*_22_	0.1457	*X*_32_	0.1457	*X*_42_	0.0475
*X*_13_	0.1354	*X*_23_	0.1068	*X*_33_	0.3547	*X*_43_	0.0241

## Data Availability

The labeled dataset used to support the findings of this study is available from the corresponding author upon request.
